# Healthcare Access for Older Indigenous Women in Latin America: A Scoping Review of Barriers, Facilitators, and Knowledge Gaps

**DOI:** 10.3390/nursrep16080282

**Published:** 2026-08-13

**Authors:** Francisco Javier Arroyo-Cruz, Karina Isabel Casco-Gallardo, Edith Araceli Cano-Estrada, Benjamín López-Nolasco, Abigahid Vianey Morales-Ortiz, Claudia Atala Trejo-García, José Antonio Guerrero-Solano

**Affiliations:** Academic Area of Nursing, Tlahuelilpan Superior School Campus, Autonomous University of the State of Hidalgo, Tlahuelilpan 42780, Hidalgo, Mexico; francisco_arroyo@uaeh.edu.mx (F.J.A.-C.); edith_cano@uaeh.edu.mx (E.A.C.-E.); benjamin_lopez8496@uaeh.edu.mx (B.L.-N.); abigahid_morales@uaeh.edu.mx (A.V.M.-O.); ctrejo@uaeh.edu.mx (C.A.T.-G.)

**Keywords:** healthcare, health services research, older adults, indigenous people, Latin America, dimensions, barriers, facilitators

## Abstract

**Background**: Over recent decades, Latin America has undergone significant population aging, accompanied by persistent inequalities that adversely affect healthcare access, particularly among Indigenous populations. This scoping review aimed to map and synthesize the available evidence on healthcare access for older Indigenous women in the region, identifying relevant dimensions, barriers, facilitators, and existing knowledge gaps. **Methods**: A systematic search was performed in scientific databases and gray literature up to 19 June 2026, including studies published between 2020 and 2026 in Spanish, English, or Portuguese. The PCC framework (Population, Concept, Context) was applied. Following a multi-reviewer selection process involving seven reviewers and data extraction using a standardized matrix, the findings were narratively synthesized in accordance with the PRISMA-ScR guidelines. **Results**: Eleven sources of evidence were included, representing six Latin American countries and including one regional review; qualitative approaches were the most common among the primary studies. Eight dimensions of accessibility were identified: geographic, economic, cultural, organizational, linguistic, functional, administrative, and social. Principal barriers included geographic isolation, poverty, a lack of cultural and linguistic relevance, bureaucratic hurdles, structural racism, and a scarcity of disaggregated data. Facilitators encompassed family and community networks, traditional medicine, local leadership, social programs, and technological/intercultural adaptations such as telemedicine. Compared with the Levesque framework, approachability, appropriateness, and formal system-navigation mechanisms were not clearly represented in the Latin American evidence, while engagement was insufficiently characterized. **Conclusions**: The findings indicate that structural and cultural barriers continue to persist.

## 1. Introduction

In recent decades, Latin America has undergone demographic changes characterized by population aging, driven by declining fertility rates and increased life expectancy [1]. The growth of the older adult population has generated a progressive demand for healthcare services oriented toward the care of this population [2]. This situation is reflected in a context characterized by significant social and economic inequality, which directly interferes with opportunities for healthcare access [3]. In the Region of the Americas, timely, continuous, and high-quality access to healthcare services remains a challenge today, with particular emphasis on population groups experiencing structural conditions of vulnerability [4].

From a general perspective, Indigenous peoples in Latin America have been historically affected by phenomena such as social exclusion, poverty, and discrimination; this becomes evident when observing gaps in health indicators [5]. While it is true that there are regulatory advances, as well as efforts to implement equity-oriented policies, the Indigenous population in the Latin American region still faces barriers that limit accessibility to healthcare services; among these, geographic dispersion of communities, economic limitations, linguistic barriers, a lack of cultural appropriateness in care, and experiences of discrimination within healthcare systems can be mentioned [4,6,7]. These inequalities are embedded within a broader framework of social determinants of health, which, when present, condition well-being throughout a person’s life course.

When addressing the Indigenous population, it becomes essential to consider older women, as they represent a particularly vulnerable group involving multiple axes of inequality related to age, gender, and ethnicity [8,9]. From this preamble, many Indigenous women have experienced limited access to formal education as well as formal employment, thereby generating unequal participation in social protection systems, which collectively can translate into fewer economic resources and greater dependency in old age [10]. To this can be added the inequalities derived from aging itself, such as the increase in chronic diseases and a greater need for continuous care, as well as the cultural and linguistic barriers that hinder interaction with healthcare services [11].

Addressing this intersecting set of disadvantages reveals scenarios of intersectional vulnerability that directly affect accessibility to healthcare among older Indigenous women. In this sense, intersectionality refers to the inequality in aging and health decline, which do not occur due to chronological age in isolation, but rather through how age interacts in a multiplicative manner with ethnicity and gender [12]. In addition to this, contemporary structural racism represents a colonial legacy that devalues the knowledge systems and bodies of Indigenous women, negatively affecting their treatment within the healthcare system. Therefore, current structural racism demands the rejection of traditional healthcare approaches and the adoption of a decolonial lens that addresses power asymmetries in healthcare delivery [13].

The concept of “accessibility to care” is grounded in a community nursing perspective, which defines access as a dynamic process of interaction between the service user and the healthcare system [6,10,14]. In this context, healthcare personnel play a central role, particularly at the primary and community care levels, serving as facilitators of continuity of care and promoting culturally appropriate care practices [9].

This concept encompasses geographic, economic, organizational, and cultural components that shape equity in care. Within this framework, accessibility is a central element to guaranteeing the right to health among older women. In this setting, primary and community care nursing personnel play a direct facilitating role, applying transcultural care theory [15] to mitigate environmental barriers, ensure continuity of care, and promote culturally congruent care practices.

Based on what has been mentioned, it becomes imperative to have a robust understanding of the barriers, as well as the facilitators of healthcare access in older Indigenous women. This understanding is key for decision-making regarding policies aimed at eliminating or limiting barriers, as well as enhancing accessibility facilitators [5,7].

The present scoping review is highly relevant given its focus, as scientific evidence on healthcare access among older Indigenous women in Latin America remains scattered and shows notable heterogeneity in methodological approaches and study contexts. Although primary research exists that addresses the phenomenon of healthcare access in Indigenous populations, in women, or in older adults, syntheses that integrate these axes jointly from a regional perspective are limited [4,6].

In this regard, a review study is necessary to identify, describe, and analyze the available evidence on accessibility to healthcare services in older Indigenous women in Latin America, with the purpose of providing relevant inputs for nursing practice, research, and the design of public policies oriented toward health equity [7]. This study aims to map and synthesize the available scientific evidence on access to healthcare services among older Indigenous women in Latin America, using a scoping review to identify dimensions of accessibility, barriers, facilitators, and knowledge gaps.

## 2. Materials and Methods

To formulate the review question for this study, the PCC framework comprised older Indigenous women as the population, healthcare access as the concept, and Latin America as the context.

The review question was established as: What evidence exists regarding accessibility to healthcare services among older Indigenous women in Latin America? A scoping review was conducted to identify and synthesize the scientific evidence on the proposed topic—namely, accessibility to healthcare services among older Indigenous women in Latin America. This type of study is appropriate when the evidence is heterogeneous, scattered, and limited in methodological scope; furthermore, it allows for the mapping of the main findings, approaches, and existing knowledge gaps within a specific field of study. The review process was conducted in accordance with the PRISMA-ScR guidelines for scoping reviews [16]. This review was registered on OSF with the associated project number: https://osf.io/wxzfh (accessed on 19 June 2026) [17].

It is important to acknowledge certain deviations between the registered protocol on OSF and this final manuscript. Firstly, the title registered on OSF differed slightly from the current manuscript title. Initially, the project aimed to focus specifically on ‘Structural and Social Determinants’ of healthcare access. However, during the refinement of the study protocol, it became evident that a more comprehensive review required a broader scope, encompassing all ‘Dimensions, Barriers, and Facilitators’ of access to health services. This adjustment was made to accurately reflect the comprehensive nature of the evidence identified and discussed. Secondly, the OSF registration protocol included two authors (S.A. Mendoza-Mojica and A. Maya-Sánchez) who do not appear in the final author list of this manuscript. These individuals decided to withdraw from the study before the methodological process, including the search and selection of evidence, was initiated. Consequently, they did not contribute to the execution of the review or the writing of the manuscript, and thus are not listed as authors in this final publication. The authors listed herein are those who actively contributed to and are responsible for the entirety of the study.

### 2.1. Search Strategy

To ensure a comprehensive and in-depth mapping of the diverse, heterogeneous, and potentially scattered evidence on healthcare service accessibility among older Indigenous women in Latin America, a broad range of databases and gray literature sources were systematically selected. This extensive approach was critical given our specific geographic and population focus, as relevant research, particularly on Indigenous communities in Latin America, often resides in regional databases or non-traditional publications. Therefore, major international databases were complemented with specialized regional portals and comprehensive gray literature searches to capture all pertinent information.

A systematic search was conducted in PubMed, Scopus, Cochrane Library, Embase, MedRxiv, DOAJ, Wiley Online Library, Redalyc, WHO IRIS, Dialnet, the National Repository of Mexico, Biblioteca Digital de São Paulo, TESEO, LA Referencia, the BVS Regional Portal, LILACS, and Google Scholar. Additionally, a gray literature search was executed in open-access and preprint repositories, university repositories, repositories of international organizations and government health agencies, specialized gray literature sources, and academic search engines. The search strategy combined descriptors in Spanish (DeCS) and English (MeSH), as well as free-text terms related to the population, the phenomenon of interest, and the geographic context.

The main terms used included: mujeres indígenas, pueblos indígenas, persona mayor, envejecimiento, accesibilidad a los servicios de salud, acceso a la atención de salud, and América Latina, as well as their equivalents in English and Portuguese. The Boolean operators and OR were employed to combine the search terms. The strategy was adapted to the specific characteristics of each database, and the final search was executed on 19 June 2026.

### 2.2. Search Strategy (Query String)

((“indigenous peoples”[All Fields] OR “indigenous”[All Fields] OR “aboriginal”[All Fields] OR “native peoples”[All Fields]) AND (“women”[All Fields] OR “female”[All Fields]) AND (“older adults”[All Fields] OR “elderly”[All Fields] OR “aging”[All Fields] OR “aged”[All Fields]) AND (“accessibility”[All Fields] OR “access to health services”[All Fields] OR “healthcare access”[All Fields] OR “availability of health services”[All Fields] OR “barriers”[All Fields] OR “facilitators”[All Fields]) AND (“Latin America”[All Fields] OR “South America”[All Fields] OR “Central America”[All Fields])).

The provided PubMed query string serves as a comprehensive example of our strategy. It is important to note that this strategy was adapted for each database, considering its unique indexing and syntax. For instance, while [All Fields] is used in PubMed for broad term searching, the equivalent in Scopus typically uses ALL(keyword) or TITLE-ABS-KEY(keyword), and in Embase (via Ovid), it often translates to .mp. (multi-purpose field), as shown in these illustrative examples:

- PubMed: “indigenous peoples”[All Fields]
- Scopus: ALL(“indigenous peoples”)
- Embase (Ovid): indigenous peoples.mp.

Providing the full, adapted search strings for all databases and sources would be excessively lengthy for the main manuscript. However, these comprehensive strategies have been meticulously documented for reproducibility and are available upon request.

### 2.3. Eligibility Criteria

#### 2.3.1. Inclusion Criteria

Primary studies employing qualitative, quantitative, or mixed-methods designs, as well as evidence syntheses or review studies, were eligible when they addressed accessibility or access to healthcare services, including barriers, facilitators, perceptions, experiences, interventions, programs, or related determinants of the phenomenon. For the purposes of this review, access was defined as the possibility of reaching, entering, or obtaining coverage from a physical or institutional healthcare service or setting [18], whereas accessibility referred to the characteristics and dimensions of healthcare services that facilitate or hinder such access [19].

Healthcare access or accessibility was not required to constitute the primary outcome of the study. Studies were also eligible when they provided clearly identifiable and extractable evidence on contextual, social, economic, functional, cultural, or organizational factors that could be mapped to a dimension of healthcare accessibility or to the population’s ability to perceive, seek, reach, obtain, or engage with healthcare.

Studies and reviews focusing on Indigenous women aged 60 years or older were eligible. Evidence including broader Indigenous populations was also considered when findings relevant to older Indigenous women could be clearly identified or extracted. The chronological threshold of 60 years was based on standardized criteria commonly used to define the older adult population in Latin America [20]. Nevertheless, the analysis also considered that functional aging may occur earlier in Indigenous populations as a consequence of cumulative structural inequalities across the life course [6].

Studies or reviews conducted in, or explicitly reporting evidence from, Latin American countries were included.

Publications in Spanish, English, or Portuguese were eligible.

Documents published between January 2020 and the final search date in 2026 were considered to identify contemporary evidence on healthcare access within a highly dynamic regional context [21].

#### 2.3.2. Exclusion Criteria

Editorials, letters to the editor, comments, opinions.

Studies that neither directly assessed healthcare access or accessibility nor provided identifiable and extractable findings related to barriers, facilitators, determinants, service-use conditions, or population capabilities relevant to obtaining healthcare.

Research conducted outside the Latin American context.

Studies that did not allow for the identification of specific results for older Indigenous women.

#### 2.3.3. Study Selection Process

The identification, screening, eligibility, and inclusion process was conducted in accordance with the PRISMA-ScR guidelines [16].

An initial screening based on titles and metadata was conducted directly within the databases, repositories, and academic search engines. At this stage, all 2401 identified records were manually screened by the review team against the Population, Concept, and Context (PCC) criteria. Records that were clearly unrelated to the review question based on available title and metadata information were excluded at this stage (n = 2310). The remaining 91 potentially relevant records were exported to the Zotero reference manager for full abstract and full-text evaluation. No automation tools were used for record screening, study exclusion, or eligibility decisions; all decisions were made by human reviewers.

To ensure consistency in the application of the eligibility criteria, the review team conducted a calibration exercise before formal screening. During this exercise, all seven reviewers independently screened the same sample of records and compared their decisions to clarify the application of the predefined eligibility criteria. Formal screening then proceeded through three sequential stages: title assessment, abstract and keyword assessment, and full-text eligibility assessment. When disagreements occurred, decisions were finalized by majority vote among the review team, with consultation of the corresponding authors when additional methodological clarification was required.

#### 2.3.4. Data Extraction and Analysis

Data extraction was carried out using a matrix designed specifically for this review.

The data extraction matrix was developed by the review team, guided by the PCC framework and the review objectives. Its content was meticulously defined, clearly outlining all variables to be extracted, including bibliographic characteristics (author, year, country), population details (ethnic group, age, language, educational level, sociodemographic situation), methodological design, type of healthcare service, and the primary findings related to accessibility dimensions, barriers, and facilitators. Comprehensive, standardized instructions were provided to all independent extractors to ensure a consistent approach. A pilot extraction on a subset of studies was conducted to refine the matrix and ensure a shared understanding among extractors, thereby ensuring its consistent and reliable application.

Data extraction was conducted using the standardized matrix described above. AI-assisted tools were used only after study selection to support the preliminary organization of extracted information. No AI-generated output was accepted as final data. Human reviewers verified each extracted item against the original source and completed the final extraction matrix. Any discrepancy between AI-assisted organization and human assessment was resolved by reference to the original publication. AI was not used for record screening, study exclusion, eligibility decisions, or autonomous interpretation of the findings. Data were subsequently synthesized narratively into thematic categories aligned with the dimensions of healthcare accessibility.

Consistent with the methodological framework of a scoping review, which aims to map the breadth of existing evidence irrespective of its methodological quality, an assessment of the risk of bias in the included studies was not performed. This deliberate choice allows for a comprehensive overview of the diverse landscape of research, facilitating the identification of all relevant studies and knowledge gaps within the field.

#### 2.3.5. Ethical Considerations

As this study is a review of published literature, it did not require approval from a research ethics committee. Nevertheless, the principles of scientific rigor, transparency, and correct citation of sources were respected.

## 3. Results

### 3.1. Study Selection

**Figure 1:**
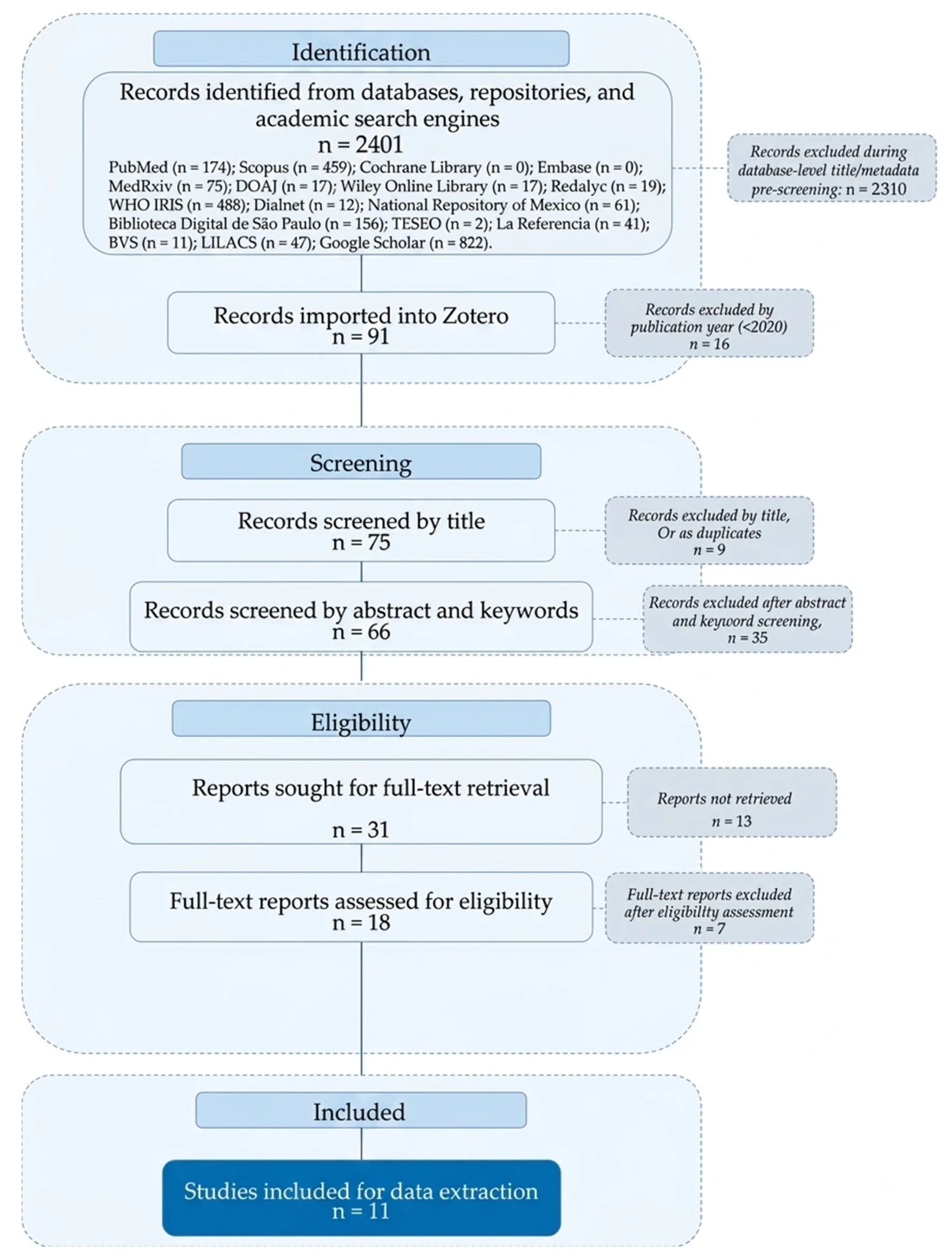
PRISMA ScR flow diagram of the study selection process.

A total of 2401 records were identified through database, repository, and academic search engine searches. All identified records underwent an initial title- and metadata-based screening directly within the respective information sources. At this stage, 2310 records that were clearly unrelated to the Population, Concept, and Context (PCC) criteria were excluded, and 91 potentially relevant records were exported to Zotero (version 9.0.6) for subsequent screening. Of these, 16 records published outside the predefined period were excluded, leaving 75 records for further title screening. Nine additional records were removed because of duplication or failure to meet the eligibility criteria, leaving 66 records for abstract and keyword screening. Following abstract and keyword screening, 35 records were excluded, and 31 reports were sought for full-text retrieval. Thirteen reports could not be retrieved, leaving 18 full-text reports for eligibility assessment. Seven full-text reports were excluded according to the predefined eligibility criteria, resulting in 11 studies included in the scoping review. The complete study selection process is presented in Figure 1.

The records were identified from PubMed (n = 174), Scopus (n = 459), Cochrane Library (n = 0), Embase (n = 0), MedRxiv (n = 75), DOAJ (n = 17), Wiley Online Library (n = 17), Redalyc (n = 19), WHO IRIS (n = 488), Dialnet (n = 12), the National Repository of Mexico (n = 61), Biblioteca Digital de São Paulo (n = 156), TESEO (n = 2), LA Referencia (n = 41), BVS (n = 11), LILACS (n = 47), and Google Scholar (n = 822).

The study selection process is shown in Figure 1.

### 3.2. General Characteristics of Evidence

The detailed characteristics of the included sources of evidence are presented in Supplementary Table S1, provided as a separate Supplementary File S1.

The review identified eleven eligible sources of evidence published between 2020 and 2026 [22,23,24,25,26,27,28,29,30,31,32], representing evidence from six Latin American countries—Mexico [22,24,27,29], Colombia [23,30,31], Chile [26,30,32], Costa Rica [25], Argentina [30] and Brazil [30], as well as one regional documentary source covering Latin America and the Caribbean [28]. Qualitative studies predominated [23,24,27,29], primarily utilizing ethnographic [23,26,29], narrative [22], descriptive [25,26] and participatory action research [23] designs, although literature reviews [30], quantitative studies [32], and telemedicine-based interventions [31] were also represented.

Because healthcare access was not the primary outcome in all included sources, the synthesis distinguished direct evidence from contextual evidence. Sources [26,30,32] were retained because they provided extractable information on functional, socioeconomic, environmental, or social conditions relevant to healthcare accessibility or to the ability to seek, reach, obtain, or remain engaged with care. These findings were treated as contextual evidence and were not interpreted as direct measures of healthcare access or service utilization. When sex-disaggregated findings were unavailable, the findings were not interpreted as women-specific estimates.

The evidence included diverse Indigenous peoples, including Mixteco [29], Purépecha [22,24], Mazahua [22,24], Maya [27,28], Ngäbe-Buglé [25], Awá [23], Mapuche [26,28,32], Aymara [28,32], Karmata Rúa (Embera-Chamí) [31], and indigenous communities from Argentina, Brazil, Colombia, and Chile [30]. The healthcare services analyzed spanned primary care [23,25,31], second- and third-level hospitals [22], rural medical units [27], intercultural care models [23], telemedicine programs [31], and, in some sources, interactions between biomedical services and traditional Indigenous medicine [22,23,28].

Participants were, for the most part, Indigenous women aged 60 years or older residing in rural communities or urban settlements with socially marginalized conditions [29,32]. Consistently, the studies described low education levels [22,24,27,29,30,32], a predominance of informal or unpaid work [22,25,29], limited income [22,24,29], scarce social security coverage [22,24], and partial reliance on family support or government programs [24,27]. Nonetheless, some studies included an Indigenous population of both sexes or did not present results disaggregated by sex or age [26,32].

### 3.3. Dimensions of Accessibility Identified in the Literature

The analysis of the studies identified eight dimensions that, in interrelated ways, relate to the accessibility of older Indigenous women to healthcare services in Latin America. Although the studies employed distinct conceptual frameworks and research objectives, common dimensions emerged across studies: service availability, socioeconomic conditions, healthcare system organization, and the cultural particularities of Indigenous communities.

Geographical accessibility was one of the most recurrent dimensions. Included sources described long distances to healthcare facilities, transportation limitations, territorial dispersion, and the need to travel outside the community to obtain care [22,23,25,27,31]. These barriers can be particularly restrictive in older age because mobility limitations and dependence on others for transportation may reduce the ability to reach healthcare services.

The economic dimension was closely linked to structural poverty. Included sources reported low income, lack of social protection, transportation costs, and economic dependence as conditions that can limit the ability to seek and obtain healthcare [22,24,25,27,29].

Regarding cultural accessibility, the literature indicates that healthcare delivery continues to struggle to incorporate Indigenous worldviews, traditional care practices, and community ways of understanding health and illness [23,24,27,28]. Various authors noted that the scarce cultural relevance of services favors a detachment between Indigenous women and the healthcare system, a situation described in research conducted with Mapuche [26,28,32], Mixtecas [29], Mayas [27,28], and other Indigenous peoples.

Organizational accessibility was related to the characteristics of the healthcare services [22,25,27]. The studies reported insufficient staffing, limited medication availability, inadequate infrastructure, restricted operating hours, prolonged waiting times, and difficulties with referrals between levels of care [22,23,25,27]. These conditions were consistently described in research conducted in primary care services across various Latin American countries [23,25,27].

Linguistic accessibility was observed mainly in communities where an Indigenous language constitutes the primary medium of communication [22,27,28]. Some studies have noted that the absence of bilingual staff or adequate translation mechanisms hinders communication between users and healthcare professionals, leading to misunderstandings, reduced comprehension of therapeutic instructions, and the perception of care that is disrespectful of cultural differences [22,23,27].

Functional accessibility was related to conditions inherent to aging [22,25,27]. Various studies described mobility limitations, dependence on transportation, visual or hearing decline, and the presence of chronic diseases—factors that increase the difficulty of attending healthcare services opportunistically, especially when geographical and economic barriers coexist [22,25,27,31].

Administrative accessibility has been less explored in the literature; however, some studies have described obstacles stemming from institutional requirements, bureaucratic processes, limited insurance coverage, and difficulties in enrolling in public health programs or services, particularly in contexts of high marginalization [22,24].

Finally, social accessibility reflected the influence of family and community networks on the ability to seek, reach, and sustain care [22,23,27,29]. Family, community, and institutional support networks can facilitate healthcare access, whereas social isolation, family migration, and the absence of caregivers may increase the vulnerability of older Indigenous women. Gallardo-Peralta et al. [32], although not designed to assess healthcare access directly, provided contextual evidence on family and social support among older Indigenous participants. These findings were interpreted as a potential enabling condition within the social dimension of accessibility rather than as a direct measure of healthcare access.

### 3.4. Identified Limitations or Barriers to Healthcare Access

Geographical isolation and the saturation of basic services emerge as central barriers, necessitating referrals to specialists located 45–60 min away and incurring associated transportation costs [25]. Long distances to health units, sporadic coverage, and the concentration of quality services in privileged areas recur as a pattern [22]. Distance and transportation costs are also documented as explicit barriers [27], alongside long waiting times and hospital care limited to one week or less per year in communal territories [23], and a lack of transportation coupled with low availability of formal services in both urban and rural areas [24].

Job insecurity, resource constraints, and migration as a subsistence strategy limit access to healthcare [29]. Informal work without social security and intra-family indebtedness to afford private consultations are identified as economic barriers [22]. Poverty and economic insecurity, with insufficient income to cover basic health needs, are equally reported [24] as are economic barriers alongside the predominance of the subsidized regime and the precariousness of the regional healthcare system.

Another relevant limitation was the presence of cultural and linguistic barriers. Several studies point out that healthcare services do not always integrate the language, knowledge, worldview, or traditional care practices of Indigenous peoples. This generates mistrust, fear, difficulty understanding medical instructions, care perceived as disrespectful, and low cultural adequacy of services [22,23,27,28].

Likewise, barriers associated with institutional invisibility and a lack of differentiated data were observed. The low availability of information disaggregated by Indigenous peoples, age, gender, or ethnic status makes it difficult to recognize the specific needs of older women and limits the formulation of relevant public responses [28].

The studies also highlight the accumulation of disadvantages across the life course, where gender, age, ethnicity, poverty, low educational attainment, racism, rurality, and social exclusion combine and deepen vulnerability in old age. This accumulation affects health, autonomy, nutrition, mobility, and effective access to services [24,29,30].

Structural racism and historically exclusionary policies are identified as root causes of the precariousness of services [22]. The neoliberal, market-oriented model of the healthcare system, along with armed conflict and public order situations, is identified as a structural obstacle to implementing intercultural models [23]. Territorial conflicts, criminalization, and militarization that restrict access to services are reported at a regional level [28]. Triple discrimination—based on being women, Indigenous, and older—is proposed as a mechanism that deepens access inequities [24]. Racism, structural discrimination, violence, and armed conflict recur as macro-structural barriers.

Additional contextual evidence described food insecurity, environmental conditions, mobility limitations, and socioeconomic vulnerability [26,30]. These findings were interpreted as contextual conditions that may shape healthcare needs and the capacity to seek or obtain care, rather than as direct measures of healthcare-access barriers.

### 3.5. Facilitators

Family and community support networks are the most frequently reported facilitators. Families, sons, daughters, grandchildren, partners, and neighborhood networks provide transportation, accompaniment, daily care, medication purchases, and emotional support. Among older adults, these networks are fundamental to maintaining health in the face of limitations in the formal healthcare system. This suggests that the family network acts as the primary compensatory mechanism against structural barriers to healthcare access [27,32].

Another important facilitator identified was knowledge of traditional medicine and cultural care. This includes the presence of local healers [27]; the central role of traditional medicine and older adults as traditional healers during COVID-19 [28]; bilingual/bicultural Indigenous health agents acting as an intercultural bridge [23]; the recognition of the *jaibaná* and ancestral knowledge [31]; and cultural traditions, spirituality, and cultural expressions (songs, music, dance) acting as resilience mechanisms [25]. These serve as care resources and promote cultural continuity, especially in contexts where biomedical care is limited or culturally irrelevant.

Community participation and local leadership were also identified as facilitating elements. The included studies show that the participation of Indigenous leaders, community health promoters, wise elders (*sabios*), older adults, traditional healers, and local organizations strengthens trust and improves the relevance of health interventions [23,28,31].

In some cases, social programs and government assistance partially facilitated access to basic resources. Non-contributory old-age pensions, financial support, social protection programs, day centers (*centros de día*), and public health insurance function as support mechanisms for nutrition, medical expenses, or general subsistence, although they do not resolve structural inequalities [24,27,29].

The technological and cultural adaptation of services also emerged as a facilitator. For instance, the SmartCare project implements community telemedicine, remote monitoring, low-cost medical devices, training conducted in both Spanish and Embera-Chamí, co-design with community leaders, and recognition of ancestral practices, which can improve continuity of care in isolated communities [31].

Lastly, older women’s social organization and capacity for action emerge as significant facilitating factors. Despite conditions of poverty, discrimination, and exclusion, older women maintain strategies of resilience, work, caregiving, knowledge transmission, community participation, and mutual support [26,29]. Their experience, wisdom, and leadership are important resources for maintaining family and community health and for building more intercultural, context-specific, and equitable models of care [23,28].

## 4. Discussion

### 4.1. Overview of Identified Evidence

This scoping review aimed to map and synthesize the evidence regarding healthcare service accessibility among older Indigenous women in Latin America. The evidence was extracted from 11 studies published between 2020 and 2026. The findings confirm the persistence of structural, operational, and cultural barriers, while simultaneously highlighting the relevance of social and organizational facilitators. The studies included demonstrated notable heterogeneity in their methodological approaches, albeit with a clear predominance of qualitative designs.

In light of this, a methodological approach that combines and expands upon existing frameworks is recommended. On one hand, generating more quantitative evidence is advisable. Although qualitative studies are excellent for exploring in-depth perceptions and experiences, quantitative evidence is necessary to quantify the prevalence of barriers and facilitators. Furthermore, it is necessary to measure the impact of interventions or programs at a larger scale and generate comparable data to evaluate access across diverse regions or Indigenous groups.

A crucial finding of this review is the identification of an extremely limited number of relevant studies within the established period (11 studies), despite the use of a comprehensive search strategy across multiple databases and gray literature sources. This highlights a profound fragmentation and a remarkably scarce body of scientific evidence regarding accessibility to healthcare services for older Indigenous women in Latin America. This reality not only confirms the initial premise of our study concerning the dispersion and heterogeneity of the available evidence but also validates the pressing need for a synthesis such as the present review to make this gap visible and to stimulate future research.

Our findings reveal that the 11 identified studies cover only a fraction of the countries with significant Indigenous populations (Mexico, Colombia, Chile, Costa Rica, Argentina, and Brazil), and within these countries, only a limited number of specific Indigenous peoples (e.g., Mixtec, Purépecha, Maya, and Mapuche). To contextualize the scarcity of studies, it is known, according to various sources, that there are between 400 and 800 Indigenous peoples in Latin America, depending on the definition and source utilized [33,34,35].

Considering that Latin America harbors an immense cultural wealth, manifested in hundreds of Indigenous peoples with diverse worldviews, languages, and socioeconomic realities, the number of studies is minimal. The countries represented (Mexico, Colombia, Chile, Costa Rica, Argentina, and Brazil) are only a fraction of those with significant Indigenous populations, and within them, very few specific peoples are covered (for example, Mixteco, Purépecha, Maya, Mapuche). This leaves a vast research ‘silence’ regarding the majority of the region’s Indigenous populations and suggests that the experiences of a large number of communities and specific ethnic groups remain largely unexplored or invisible in the reviewed scientific literature. In the context of this research, this has significant implications, as the absence of evidence hinders an in-depth understanding of the needs of this vulnerable group and, consequently, the formulation and adaptation of culturally relevant and equitable health policies and programs. The scarcity of studies could be attributed to several complex factors, including: (a) the historical marginalization of Indigenous populations within research agendas, (b) methodological and ethical challenges inherent to research in Indigenous communities, (c) linguistic and cultural barriers that may hinder the participation of external researchers, (d) the lack of specific funding for this type of research in the region, and (e) the complexity of addressing an intersectional phenomenon (age, gender, ethnicity).

The scarcity of studies of this nature is not exclusive to Latin America. In low- and middle-income settings outside the region, evidence also documents substantial barriers to healthcare access among older Indigenous adults. For example, in Bangladesh, geographic isolation, cost, linguistic barriers, and mobility limitations have been reported [36]. Even in high-income countries, specific subgroups remain understudied despite a comparatively larger body of evidence [37]. Among older Indigenous women in wealthy countries, only three studies met the eligibility criteria in a digital review [37] and in the U.S., older Indigenous adults have long been excluded from health reform debates and studies [38]. Consequently, the difference lies in the fact that, in Latin America, the available evidence appears more scattered, less indexed, and less specific to older Indigenous individuals, particularly older women.

This review found that older Indigenous women most frequently access rural primary care, secondary and tertiary hospitals, and intercultural or telemedicine services, with varying incorporation of traditional Indigenous medicine. Globally, older Indigenous peoples tend to have greater access to community primary care, cultural/traditional services, and telehealth, although marked regional differences persist [39,40,41].

The strongest evidence comes from North America and, primarily, Oceania (Australia), where research highlights community-based primary care, culturally safe models, and digital health, whereas evidence from Asia, Latin America, and Africa remains limited and less focused on older women [42,43].

In North America, access is generally better when services are Indigenous-led or locally tailored and include outreach, home care, transportation support, multidisciplinary coordination, and culturally safe spaces; traditional healing also remains an important component of care, although urban access to traditional medicines is often limited [42,44].

In Canada, older Indigenous women identified availability, accessibility, affordability, recognition of Elders, and experiences of racism and discrimination as key issues, while national data show lower access to regular healthcare providers and greater unmet needs than among non-Indigenous women [40,45].

Evidence from Australia emphasizes the expansion of digital health and telehealth, but also the lack of services specifically designed for older Aboriginal and Torres Strait Islander women, who seek culturally sensitive and practical digital resources [37,41,46].

In contrast, Latin America remains underrepresented, with scarce research specifically addressing older Indigenous women. Similarly, evidence from Africa is limited, although some studies suggest that integrating traditional medicine into formal health systems could improve inclusion and access [47,48,49]. Overall, despite the heterogeneity of the evidence, rural primary care and traditional Indigenous medicine appear to constitute the main points of healthcare access for older Indigenous women in Latin America, while intercultural models and telemedicine provide limited complementary support. Access to specialized care remains markedly restricted, likely reflecting persistent structural, economic, and cultural barriers discussed below.

This review also found that the included populations were characterized by low educational attainment, predominantly informal or unpaid work, limited income, scarce social security coverage, and partial reliance on family support or government programs. Although the magnitude varies across countries, territories, and Indigenous peoples, the evidence consistently indicates significant socioeconomic disadvantage among older Indigenous women in Latin America. Indigenous populations generally experience lower access to education, services, and social protection than non-Indigenous populations [6,50]. In Mexico, women living in municipalities with a high proportion of Indigenous population were poorer, less educated, and more likely to live in rural areas [50]. These inequalities accumulate in later life, and regional analyses identify older women as one of the most economically and educationally disadvantaged groups [51].

In Brazil, moderate or severe food insecurity was more common in households headed by older adults with Indigenous self-identification, low educational attainment, and very low income. The OECD has also reported that widespread labor informality in the region restricts access to social protection and disproportionately affects women and older adults [52]. Similar patterns have been described among marginalized Indigenous women in peri-urban Bolivia, who often depend on informal micro-enterprises while continuing to provide family care [53]. Much of their productive contribution may remain invisible because subsistence activities are frequently classified as informal work or economic inactivity. These disadvantages are best understood through an intersectional lens in which poverty, rurality, ethnicity, and gender interact [54]. Historical marginalization and systemic discrimination further contribute to poorer housing, employment conditions, and baseline access to basic and health services [6,55].

### 4.2. Dimensions of Accessibility

The analysis allowed for the identification of several dimensions of accessibility to healthcare services within the mapped population: geographic, economic, cultural, organizational, linguistic, functional, administrative, and social accessibility.

The geographic dimension consistently manifests as distance, territorial dispersion, inadequate transportation, and reliance on referrals to urban or metropolitan centers [56,57,58]. Studies accurately point out that, in this population, the geographic factor is not merely “remoteness”; it interacts with age, reduced mobility, and frailty, which decrease the actual capacity to reach the service to a greater extent than in the general adult population [59,60]. Therefore, it is advisable to better distinguish between dimensions of accessibility and user capabilities.

The economic dimension encompasses direct and indirect expenses: transportation, food, accompaniment, lost income, and opportunity costs. In rural and Indigenous contexts of the Americas, these costs are compounded by structural poverty and lower labor and family flexibility to travel [42,56,60].

The cultural dimension is central in Latin America. Evidence from Guyana and Peru shows that the use of traditional medicine, ethnic trust, and the intercultural skills of healthcare staff are constitutive elements of access rather than peripheral aspects [56]. The global literature on Indigenous women also shows that “top-down” programs render services culturally hostile and reduce their utilization [61].

The linguistic dimension is also present, although likely underestimated in Latin America. Global frameworks recognize that, in practice, linguistic barriers restrict access even when the formal right exists [60]. In rural Indigenous peoples, inadequate communication with healthcare staff and low health literacy limit the comprehension of medical indications, follow-up care, and trust [36,56].

The organizational/administrative dimension includes institutional fragmentation, poor coordination, staff shortages, poorly adapted operating hours, and complex bureaucratic processes [56,62]. Furthermore, in Latin America, the limited availability of human resources is compounded by weak infrastructure and insufficient supplies [56].

The social/structural dimension is likely the deepest explanatory layer. Social exclusion, discrimination, gender and family power relations, and low educational attainment affect whether older women perceive a need, seek care, reach it, and manage to sustain it [56,63,64].

Gaps exist regarding the evidence within the Latin American context. In a review of studies from other regions of the world, such as North America and Oceania, researchers developed more refined frameworks concerning trust, cultural safety, continuity, navigation, and care led by Indigenous services [62,63,65]. Outside of Latin America, the primary conceptual expansion originates from the Levesque framework [60], which adds five dimensions on the system side—approachability, acceptability, availability/accommodation, affordability, and appropriateness—alongside five “abilities” on the population side: the ability to perceive, to seek, to reach, to pay, and to engage. Within the studies conducted in Latin America, the dimension of approachability was not identified, which entails that the person knows the service exists, understands its purpose, and perceives it as a real option. Similarly, appropriateness was not reported in the studies, a concept that goes beyond availability and acceptability to evaluate technical and interpersonal quality, coordination, continuity, and the alignment between the need and the service response [60]. The third expansion involves cultural safety as a quasi-autonomous dimension. In Indigenous adolescents from Australia, it was explicitly added as a standard dimension, differentiating it from other classical domains [66]. Furthermore, in Canada, equity-oriented care is broken down into inequity-responsive care, culturally safe care, trauma- and violence-informed care, and contextualized care. In Latin America, interculturality is heavily discussed, but less emphasis is placed on trauma-informed care or structural violence as operational dimensions of access [63]. A fourth dimension not found was system navigation. In Canada, Indigenous patient navigators, community workers, and Elders facilitate communication, referrals, and access to traditional healing [67]. In Australia, Aboriginal Health Workers fulfill similar roles: coordinating appointments, explaining indications, conducting follow-up, and resolving transportation issues [62]. In Latin America, this function appears more diffusely within “coordination” or “accompaniment” but is less formalized as an independent dimension. Consequently, the most critical open question for Latin America is whether the dimensions already described are sufficient to capture the experience of older Indigenous women, or if it is necessary to explicitly incorporate navigation, trauma, and engagement among those previously mentioned as distinct dimensions of accessibility in Indigenous old age.

### 4.3. Barriers and Facilitators of Access to Healthcare Services

The barriers to healthcare access among older Indigenous women in Latin America are complex and persistent. Our findings confirm the existence of structural, economic, cultural, linguistic, geographic, organizational, and social obstacles that significantly limit the capacity of these women to receive the care they need. These interconnected dimensions not only hinder physical access but also erode the quality, timeliness, and continuity of care, as well as trust and the cultural appropriateness of available services. Although many regional studies do not isolate older women specifically, the literature on Indigenous women in Latin America and on Indigenous aging in Mexico and other contexts shows that age adds disadvantages due to multimorbidity, reduced mobility, economic dependence, caregiving responsibilities, and cumulative trajectories of discrimination [6,54,68].

These interconnected dimensions are associated with lower utilization of preventive, diagnostic, and therapeutic services; greater delays in seeking care; poorer care experiences; and a higher probability of unmet needs [56,68]. Among Latin American Indigenous women, the observed consequences include a greater burden of infections and chronic diseases, as well as lower coverage for screenings such as mammography, with persistent ethnic gaps [50,69]. The review on Indigenous aging shows that access to services, transportation, finances, and cultural safety remain central obstacles to “aging well,” alongside the legacies of colonization, language loss, and historical trauma [68].

Barriers persist because they are anchored in distal and proximal mechanisms. Work from Canada and global health literature concur that colonialism, racism, and social exclusion produce inequalities in income, education, employment, infrastructure, and political participation, and these inequalities subsequently translate into distance, staff shortages, low quality, mistrust, and poor cultural adequacy [64,70]. The situation in other regions of the world is similar in its root causes, although some contexts have developed more institutional responses. In Bangladesh, older Indigenous individuals face discrimination, geographical isolation, prohibitive costs, linguistic barriers, limited mobility, and living apart from their children, which delays care until critical stages [36]. In Canada, Australia, New Zealand, and the United States, evidence also describes geography, stigma, racism, poor communication, a lack of culturally safe options, and mistrust of the system. The primary difference is not the absence of inequality, but rather the greater presence of explicit models of cultural safety, adapted telehealth, the integration of Indigenous healing, and the formal participation of Indigenous leaders, Elders, and workers in service design [64,71,72,73].

Despite the significant barriers, the study also identified several facilitating elements that improve access to healthcare for older Indigenous women. These include community participation and local leadership, support from social programs and government assistance, the technological and cultural adaptation of services, and the crucial social organization and capacity for action of older women themselves. These factors not only help mitigate existing challenges but also strengthen the relevance and continuity of care within these communities, although evidence regarding quantitative impact remains more limited than evidence on barriers. An example of the effect of these facilitators is that in Latin America, community participation, local leadership, and ownership by Indigenous women improve program acceptability, emergency detection, referrals, and community empowerment [74,75]. Outside of Latin America, additional facilitators emerge that are less consolidated within the region. Prominent among these are culturally adapted telehealth, models featuring an initial in-person consultation followed by remote monitoring, formal access to Elders and Indigenous healing within primary care, HPV self-sampling, and specific support policies for menopause, informal caregiving, and mental health across the life course [71,76,77].

### 4.4. Perspectives

The issue should not be analyzed merely as a sum of “barriers to access,” but rather as the result of cumulative exclusion regimes where age, gender, ethnicity, territory, and poverty interact. This perspective shifts the focus from the “adaptation” of women to the system toward the transformation of the system through structural reforms, cultural safety, stable financing, decentralization, the respectful integration of Indigenous knowledge, and community co-governance [6,78]. For older Indigenous women, the literature suggests prioritizing community-based and intercultural models, transportation and accompaniment support, culturally safe mobile or remote services, the inclusion of traditional healers, midwives, Elders, and Indigenous community health workers, as well as specific strategies for aging conditions, menopause, dementia, mental health, and multimorbidity [79,80,81]. Finally, there is a methodological perspective regarding the lack of research specifically centered on older Indigenous women in Latin America. Regional evidence is concentrated on maternal and reproductive health, among other areas, whereas aging needs, chronic diseases, functional decline, menopause, dementia, digital literacy, and long-term care remain understudied [6,37,47].

### 4.5. Limitations

This scoping review presents both methodological and evidence-derived limitations. As this was a scoping review, a formal appraisal of the methodological quality or risk of bias of the included studies was not performed; therefore, the findings should be interpreted with caution. Likewise, the search was restricted to publications between 2020 and 2026 and to documents in Spanish, English, and Portuguese. This specific time frame was a deliberate methodological choice to capture the most current evidence, recognizing the rapid and significant socioeconomic, political, and health policy transformations occurring in Latin American countries. These include fluctuations in income levels, changes in public policies and social welfare programs, currency devaluations, evolving political landscapes, and varying levels of implementation of intercultural health models. Studies conducted prior to 2020 might reflect contexts that have substantially changed, potentially introducing bias or presenting findings no longer representative of current accessibility challenges and facilitators for older Indigenous women. Potentially relevant studies published earlier or in Indigenous languages may have been excluded. Furthermore, the inclusion criteria privileged studies with specific results for older Indigenous women, meaning that broader investigations providing indirect evidence may have been excluded.

The primary limitation, however, lies in the scarcity and fragmentation of the available evidence. The number of identified studies is small relative to the diversity of Indigenous peoples in Latin America, highlighting significant knowledge gaps. Existing information is scattered, with limited indexing and scarce methodological standardization, making consistent comparisons difficult. In addition, many studies do not specifically analyze older Indigenous women, necessitating extrapolation of findings from broader populations. Similarly, evidence on the effectiveness of strategies to improve healthcare access remains limited and predominantly qualitative, compared to the abundant documentation of barriers to access. Finally, the intersectional nature of the phenomenon—resulting from the interaction among age, gender, ethnicity, territory, and socioeconomic conditions—poses an analytical challenge that the currently available evidence does not yet permit comprehensive addressing.

## 5. Conclusions

This scoping review has enabled the mapping and synthesis of available evidence regarding accessibility to healthcare services among older Indigenous women in Latin America, revealing persistent structural, social, and cultural barriers that significantly limit their access and quality of care. The identified evidence confirms that these inequalities are mediated by the complex interplay of factors such as colonialism, racism, poverty, and limited intercultural appropriateness within healthcare systems.

Facilitators such as community participation, local leadership, and the incorporation of ancestral practices were also highlighted, contributing to improved relevance and continuity of care, although quantitative evidence on their impact remains limited. This review further demonstrates the fragmentation and scarcity of studies specifically addressing this population, underscoring the need for more comprehensive and methodologically diverse research to expand knowledge about the unique needs and experiences of older Indigenous women.

Based on the findings of this study, this work contributes to the core functions of the nursing discipline by redefining care for older Indigenous women through an intersectional framework. This approach analyzes how social factors intersect to shape specific experiences, such as making visible the triple discrimination faced by older Indigenous women based on gender, age, and ethnicity. Consequently, nursing must transcend strictly biomedical perspectives and move toward holistic models that account for social determinants of health—such as accumulated poverty, geographic isolation, illiteracy, and structural racism—thereby strengthening intercultural and community nursing practice. Cultural safety must be enhanced, and linguistic and cultural barriers identified, positioning nursing as an intercultural bridge. This guides the discipline to connect formal clinical practice with community knowledge, creating safe and respectful environments for care delivery.

Furthermore, these findings can serve as a guide to inform nursing practice and care innovation. To guarantee access to healthcare services for older Indigenous women, transforming health systems and care practices is essential, rather than merely expecting women to adapt to structures that have historically excluded them. Given its continuous presence in primary and community care, nursing occupies a strategic position to recognize barriers, coordinate support networks, accompany care pathways, and promote culturally safe care.

Finally, this study contributes to advancing research and methodological innovation in nursing by revealing a critical scarcity of scientific literature specifically focused on this population group in Latin America. These findings invite researchers to generate and lead future projects in this area.

## Data Availability

No new data were created or analyzed in this study. Data sharing is not applicable to this article.

## Supplementary Materials

The following supporting information can be downloaded at: https://www.mdpi.com/article/10.3390/nursrep16080282/s1, Supplementary Table S1, Detailed Characteristics of Included Sources of Evidence on Healthcare Access for Older Indigenous Women; Supplementary File S1, PRISMA-ScR Checklist.

## Public Involvement Statement

No public involvement in any aspect of this research.

## Guidelines and Standards Statement

This manuscript was prepared in accordance with the Preferred Reporting Items for Systematic Reviews and Meta-Analyses extension for Scoping Reviews (PRISMA-ScR) reporting guideline.

## Use of Artificial Intelligence

Gemini Flash (Google LLC, Mountain View, CA, USA) was used to support the preliminary organization and systematization of information extracted from the included sources. In addition, Gemini Flash, through a custom Gem developed by the authors named Academic Translator for Nursing Scoping Reviews, was used to support Spanish-to-English translation and academic language adaptation of the manuscript. AI-assisted outputs were not accepted as final without human review. All extracted information was verified against the original publications, and all translated or language-edited text was reviewed by the authors to ensure accuracy, preservation of meaning, and appropriate scientific terminology. AI was not used for record screening, study exclusion, eligibility decisions, or autonomous interpretation of the findings. Any discrepancy identified during data organization or translation was resolved by consulting the original source material. The authors take full responsibility for the accuracy, interpretation, and integrity of the final manuscript.

## Abbreviations

The following abbreviations are used in this manuscript:

AI	Artificial intelligence
BVS	Virtual Health Library
CBPAR	Community-Based Participatory Action Research
DeCS	Descriptors in Spanish
MeSH	Medical Subject Headings
OSF	Open Science Framework
PCC	Population, Concept, Context
PRISMA	Preferred Reporting Items for Systematic Reviews and Meta-Analyses
PRISMA-ScR	PRISMA Extension for Scoping Reviews
SECITHI	Secretaría de Ciencia, Humanidades, Tecnología e Innovación
T2DM	Type 2 diabetes mellitus
