# Supplementary material for: Healthcare Access for Older Indigenous Women in Latin America: A Scoping Review of Barriers, Facilitators, and Knowledge Gaps
Source: Nurs Rep. 2026 Aug 13;16(8):282. doi: 10.3390/nursrep16080282 (PMC13516063; doi:10.3390/nursrep16080282)
Supplement: Supplementary file 1 [file nursrep-16-00282-s001.zip › nursrep-4461706-supplementary-Table S1.pdf]

Table S1. Summary and Characteristics of Included Studies on Healthcare Accessibility for Older Indigenous Women*.

Author	Year	Country	Ethnic group and language	Educational level	Sociodemographic situation	Objective	Methodological design and sample size (n)	Instruments	Type of health service	Barriers	Facilitators	Limitations
**Morales-López et al. [29]**	2022	Mexico	Indigenous women from the Mixteca region of Oaxaca.<br><br>Language: Mixtec (in its northwestern Oaxaca variant)	Generally low	It has a population of 1,474 inhabitants (51.3% women) distributed across 5 localities, with 190 inhabited houses and 53 uninhabited houses due to migration. The main economic activity is temporary subsistence agriculture, including milpa cultivation.	To understand the perspective of women and retrieve their narratives, analyzing the meanings and interpretations they express regarding poverty, with an emphasis on the relational processes of disadvantages.	Ethnographic research with a culturally situated gender perspective and a diachronic approach. Two phases (monographic and interview). (n=8).	In-depth interviews, participant observations, informal chats documented in field diaries, and direct observation in private and public spaces such as assemblies.	Not found	Lack of resources, job insecurity, and migration as a subsistence strategy.	Not found	It does not delve into the structural causes of poverty that limit healthcare access.
**Rallo et al. [25]**	2025	Costa Rica	Ngäbe-Buglé	Out of the 6 women, 5 had "no" education	High dependence on insufficient pensions, food	To explore the aging experience of	Qualitative descriptive study with semi-	Semi-structured interviews	They received primary care through the EBAIS	Geographical isolation of La Casona, saturation	Mentions proposals, not existing	Not specified.

Author	Year	Country	Ethnic group and language	Educational level	Sociodemographic situation	Objective	Methodological design and sample size (n)	Instruments	Type of health service	Barriers	Facilitators	Limitations
			Indigenous community Language: Mainly Spanish during the interviews, with interpretation into Ngäbere	and 1 completed up to 6th grade.	insecurity, and the need for financial support and infrastructure; regarding occupation: domestic work, sale of crafts, and retirement.	the Ngäbe-Buglé community and identify challenges and potential resources to improve the quality of life and the aging process of older adults in the community.	structured interviews and thematic analysis. (n= 14).		(Basic Team for Comprehensive Healthcare); for specialized services, they had to travel to hospitals.	and limitations of the EBAIS (primary care only), lack of physicians/providers, need for referrals to specialists in San Vito (45–60 min) with transportation costs, and mobility limitations of older adults that make it difficult to reach the facility.	facilitators as such.	
**Hernández Delgado and Pelcastre Villafuerte. [22]**	2026	Mexico	Purépecha (Tarasco) and Mazahua Indigenous women Language: Spanish and Purépecha	Low educational level or no formal education	Lack of basic services and adequate infrastructure, with between 25 and 38 years of urban residence. Self-employed in informal commerce. Married and widowed.	To identify the specific barriers faced by older Indigenous women in accessing healthcare, intersecting four analytical dimensions:	Qualitative study of a narrative type (n=6)	Interviews conducted in the participants' homes, recorded prior to informed consent.	Health centers, public hospitals of second and third levels, traditional Indigenous medicine practices (midwives, herbal medicine), and private consultations in neighborhood pharmacies with a	Structural barriers (supply, visibility, and geography): Limited availability and intermittent biomedical services, long distances to healthcare facilities, sporadic coverage, concentration of	The role of the researchers themselves indirectly facilitated community leaders to have contact with the services. Dependence on sons/daughter	The purposive sample is small. The focus was limited to a single settlement. Absence of the perspective of healthcare providers,

Author	Year	Country	Ethnic group and language	Educational level	Sociodemographic situation	Objective	Methodological design and sample size (n)	Instruments	Type of health service	Barriers	Facilitators	Limitations
						gender, ethnicity, age, and urban context.			community physician.	higher-quality services in privileged areas, limited transportation knowledge, family dependence, and functional limitations that restrict mobility. Historical exclusionary policies and structural racism contribute to the precariousness of healthcare access. Cultural barriers: Reliance on traditional care practices, language barriers, lack of integration of Indigenous knowledge into biomedical care, fear and mistrust of the health system,	s for transportation can also be considered a "facilitator" in the context of transportation barriers.	which reduces the transferability of the results.

Author	Year	Country	Ethnic group and language	Educational level	Sociodemographic situation	Objective	Methodological design and sample size (n)	Instruments	Type of health service	Barriers	Facilitators	Limitations
										and normalization of illness and pain due to limited access and low health literacy. Economic barriers: Informal employment, lack of social security, unstable income, and reliance on family loans to afford private healthcare. Organizational barriers: Literacy-dependent administrative processes, documentation and referral requirements, complex procedures, long waiting times, bureaucratic obstacles, indifferent		

Author	Year	Country	Ethnic group and language	Educational level	Sociodemographic situation	Objective	Methodological design and sample size (n)	Instruments	Type of health service	Barriers	Facilitators	Limitations
										treatment, and ageism in healthcare delivery.		
**Hernández-Moreno et al. [26]**	2024	Chile	Mapuche<br><br>Language: No information available	No information available.	Mapuche women over 60 years old are protagonists in food sustainability. Many face mobility and health limitations associated with age, perform subsistence labor in a context where young people migrate to the city, and partly depend on subsidies to buy food that they can no longer fully reproduce. The study does not report disaggregated quantitative data by sex regarding education, income, or household composition.	To analyze the main factors affecting the food and nutritional security of Mapuche population over 60 years old living in the rural area of Temuco.	Qualitative, descriptive, and interpretive study with ethnographic support, convenience sampling in nine Mapuche communities of Boyeco (rural Temuco); men and women ≥60 years old participated. Eleven open interviews (≈1.5 h each) and 2 focus groups (≥10 participants each, mixed) were conducted. (n=11)	Open ethnographic interviews.	No information.	Health aspects affecting food security are mentioned (pathological aging, mobility limitations, diseases related to water contamination, partial dependence on subsidies), but not barriers to healthcare service access as such.	Not mentioned.	Not mentioned.

Author	Year	Country	Ethnic group and language	Educational level	Sociodemographic situation	Objective	Methodological design and sample size (n)	Instruments	Type of health service	Barriers	Facilitators	Limitations
**Pelcastre-Villafuerte et al. [27]**	2020	Mexico	Maya Indigenous population.<br><br>Language Maya	Out of 9 female participants, the majority did not know how to read or write.	The age range of the group was 65–87 years, a breakdown by sex is not presented. They owned their homes. They lived with a partner, children, or grandchildren, and the majority identified as Catholic. They received support from their children when they were sick.	To explore the health needs perceived by older Indigenous adults in a Maya region of Mexico and analyze the extent to which available health services align with their needs.	Qualitative study conducted in two Maya communities in southeastern Mexico. Data were collected in 2013–2014 through home-based and health center semi-structured interviews<br>(n= 20) older adults, 9 traditional healers, and 7 public health providers.	Semi-structured interviews	Rural medical units with a consultation room, residence for doctors, pharmacy, and midwives/maternity, staffed by general practitioners, nurses, and interns.	Language (doctors are not fluent in Maya), distance and transportation costs, lack of medications and equipment, limited operating hours (daytime only), shortage of specialized staff, mistrust in services and uncordial treatment, reduced mobility, and illiteracy that hinders following medical indications.	Presence of local healers and family support (children help and transport them), existence of Rural Medical Units with free care, and government programs providing financial support and insurance (*PROCAMPO*, *"70 y mas "*, *"Oportunidades"*, *"Seguro Popular"*).	Not mentioned.
**Pan American Health Organization (PAHO). [28]**	2023	Latin America and the Caribbean	No specific information for older women<br><br>Language:	No specific information for older women.	Structural inequalities and violence against Indigenous women, gaps in poverty, education, health,	To analyze the current situation and projections of Indigenous	Document review	Not applicable	Not mentioned	Difficulty accessing healthcare services and a lack of culturally adequate services within	Importance and central role of traditional medicine and traditional	Not mentioned

Author	Year	Country	Ethnic group and language	Educational level	Sociodemographic situation	Objective	Methodological design and sample size (n)	Instruments	Type of health service	Barriers	Facilitators	Limitations
			Quechua, Aymara, Mapuche, Maya, Guaraní, Tzeltal, Achuar; however, they do not focus specifically on older women.		and access to pensions.	peoples in Latin America and the Caribbean in the context of aging and COVID-19.				territories. Scarce availability of information and health data disaggregated by Indigenous peoples, which hinders visibility and response. Territorial conflicts, criminalization, and militarization that restrict access to resources and healthcare services. Poverty, overcrowding, and deficiencies in water and sanitation that increase health vulnerability and limit preventive measures. Lack of staff and services with cultural relevance and	healers in community health and responses to COVID-19; proposal to strengthen traditional medicine and its agents. Community measures undertaken by Indigenous peoples led by older adults and traditional healers during the pandemic.	

Author	Year	Country	Ethnic group and language	Educational level	Sociodemographic situation	Objective	Methodological design and sample size (n)	Instruments	Type of health service	Barriers	Facilitators	Limitations
										adequate records/care.		
**Casas Cruz et al. [23]**	2022	Colombia	Awá Indigenous people<br><br>Language: Awapit (native Awá language), and some participants were bilingual in Spanish and Awapit	Not found	High mortality and morbidity-mortality rates due to malnutrition, diarrheal, and respiratory diseases that especially affect older adults. Mortality from tropical diseases.	To create a healthcare model that reduces health inequities for the Awá Indigenous population of Nariño, Colombia.	The study was guided by the Community-Based Participatory Research (CBPAR) paradigm. It was based on critical ethnography, following a cyclical methodology. Among the participants were older adults without specifying the number: traditional healers, midwives, and traditional "*sobadores*", principal governors, members of the Indigenous guard, and elder sages.<br>(n= 4, 3 healers and 1 wise elder)	Analysis of legal, health, and technical documents, participant observation, interviews with key informants, focus groups, and field diaries.	A Concerted Model of Intercultural Healthcare focused on primary healthcare and social determinants of health from the Awá worldview. Integration of ancestral Awá medicine with the healthcare model. Multidisciplinary mobile health teams Permanent inpatient and extramural care in communal territories.<br>Services based on health promotion, community empowerment,	Identified barriers to healthcare access: Cultural and trust barriers: They feel mistrust and shame when being examined. Hospital food is not culturally adapted. Logistic and geographic barriers: Long waiting times for care. Hospital medical care in communal territories is one week or less per year. Socio-environmental and security problems: Current humanitarian situation, armed conflict. Structural health model: The	Indigenous participation and leadership, intercultural bridges or Indigenous health workers (bilingual and bicultural men and women, professionally trained in health) acting as links. Dialogue and coordination concerning Western and traditional Awá medical knowledge, based on recognition, respect, and	Lack of financial resources from governments and agencies. The neoliberal and market-based model of the Colombian health system is a structural obstacle, and the concerted model runs the risk of being subordinated to this approach of economic profitability. Public order situations are

Author	Year	Country	Ethnic group and language	Educational level	Sociodemographic situation	Objective	Methodological design and sample size (n)	Instruments	Type of health service	Barriers	Facilitators	Limitations
									social participation in health, decentralized healthcare.	market-based model on which the Colombian health system is grounded is a structural obstacle that may subordinate the Indigenous health model. Public order problems: The humanitarian situation and public order issues historically experienced by the region, coinciding with Awá settlements, represent a threat to the implementation of the model.	mutual coordination. Training and capacity-building for traditional medicine agents, incentives for cultivating medicinal plants, and meetings for knowledge exchange. Continuous education for Mobile Health Teams and the community. Progress in building the Intercultural Health System of Indigenous Peoples (SISPI) as a legal, political,	a threat and a clear limitation for the process.

Author	Year	Country	Ethnic group and language	Educational level	Sociodemographic situation	Objective	Methodological design and sample size (n)	Instruments	Type of health service	Barriers	Facilitators	Limitations
											and financial-administrative framework.	
**Mindiola Lina and Ortega-Bolaños. [30]**	2022	Argentina, Brazil, Colombia, and Chile	Not mentioned<br><br>Language: Spanish, Portuguese	Low level of schooling, low schooling, not having a professional degree.	The incidence of type 2 diabetes mellitus (T2DM) was associated with aspects such as age between 65 and 74 years, and being female. Processes of migration, displacement, transition to urban areas, abandonment of traditional territories, changes habits, and a greater dependence on industrialized foods.	To compare risk factors associated with T2DM in Indigenous communities from four countries (Argentina, Brazil, Colombia, and Chile) during the period 2010-2020.	Literature review through consultation across different databases such as SciELO, PubMed, Dialnet, LILACS	Systematization matrix and the use of pre-established criteria	Not mentioned	In Brazil, displacement toward urban areas has become a crucial element toward the adoption of modifiable risk factors. Additionally, a deficit in basic sanitation structures, sedentary lifestyles, and population aging are observed socially. In the local context, the incidence of T2DM was associated with sociodemographic characteristics such as rural residence,	Indigenous communities in Brazil have sought a closer approach to urban areas in order to achieve greater survival.	Speaks broadly in the results.

Author	Year	Country	Ethnic group and language	Educational level	Sociodemographic situation	Objective	Methodological design and sample size (n)	Instruments	Type of health service	Barriers	Facilitators	Limitations
										poor accessibility to health services, use of alcohol and cigarettes, low physical activity, limitations in knowledge about the disease, and low adherence to medical recommendations for self-care.		
**Hernández-Delgado Y, Pelcastre-Villafuerte BE. [24]**	2025	Mexico	Primarily Indigenous Tarascan/P'urhépecha women originally from Michoacán; one Mazahua woman from the State of Mexico also participated. Indigenous	Participants 1, 2, 3, and 5: no formal education. Participant 4: second grade of primary school. Participant 6: primary school, unspecified grade.	*Sex and age:* all participants were older Indigenous women, aged between 58 and 83 years. *Place of residence:* lived in the El Rehilete neighborhood, Zapopan, Jalisco, an informal urban area with fewer than 120 Indigenous residents.	To analyze how social inequalities accumulate over the lifecourse and shape old age among Indigenous women living in an urban context.	*Approach:* qualitative. *Design:* narrative, based on the life histories of the participants. *Theoretical framework:* Cumulative Disadvantage Theory. **Sample:** 6 participants.	Semi-structured interview guide, designed to collect the participants' life histories. Individual interviews conducted in the women's homes.	2.46% had access to IMSS. 30.91% had access to IMSS-Prospera or IMSS-Bienestar. 1.24% had no access to healthcare services.	Lack of social security, related to precariousness and informal employment. Poverty and low income, which hindered meeting basic needs, including medical care. Linguistic and cultural barriers, as participants primarily spoke Purépecha or	Family support. Government programs, which constituted a source of financial support. The availability of institutional schemes such as IMSS and IMSS-Bienestar/IMS	Small sample size: only six Indigenous women participated. Purposive sampling: participants were not randomly selected, and thus do not statistically represent all older

Author	Year	Country	Ethnic group and language	Educational level	Sociodemographic situation	Objective	Methodological design and sample size (n)	Instruments	Type of health service	Barriers	Facilitators	Limitations
			languages: Tarascan, called P'urhépecha by its speakers, and Mazahua, whose endonym is Jñatjo.		*Ethnic identity:* five were Tarascan from Michoacán and one was Mazahua from the State of Mexico. *Length of residence in the city:* between 25 and 38 years; one participant had resided there for more than 30 years. *Marital status:* four were married and two were widowed.					Mazahua and did not speak Spanish upon arrival in the city.	S-Prospera for a portion of the population.	Indigenous women. Limited geographical scope: the study was conducted in a single neighborhood in Zapopan, Jalisco (El Rehilete), so its findings cannot necessarily be generalized to other communities or cities.
**Garcia Arias et al. [31]**	2025	Colombia	Karmatá Rúa Indigenous<br><br>Language: Spanish and Embera-Chamí.	Not reported in a sociodemographical manner, different literacy levels is mentioned.	Rural, geographically isolated Indigenous population with economic vulnerability, limited access to formal health infrastructure, and	To present and implement the SmartCare Technologies project, a community telemedicine	Translational, participatory, community-based, and user-centered research study/intervention (n= 6 households)	Participatory tools such as empathy maps, scenario building, identification of cultural preferences, mapping of local	Community telemedicine, home telemonitoring, remote monitoring of vital signs, follow-up of chronic diseases, early risk	Not found	Co-design with leaders, Elders, health workers, and traditional healers; community participation from the	Small size of the initial pilot; satellite connectivity problems; road difficulties for equipment

Author	Year	Country	Ethnic group and language	Educational level	Sociodemographic situation	Objective	Methodological design and sample size (n)	Instruments	Type of health service	Barriers	Facilitators	Limitations
					cultural barriers to receiving biomedical care. Older adults with reduced mobility, presenting chronic diseases.	model co-designed with the Karmata Rúa Indigenous community to improve healthcare access for older adults with reduced mobility through remote monitoring, low-cost medical devices, culturally adapted interfaces, and articulation between the biomedical system and ancestral		care routes, persona-journey simulations, and open dialogues were used. For evaluation, debriefings with caregivers, focus groups, patient satisfaction interviews, observational notes, Likert-type satisfaction scales.	detection, primary/community care, coordination with the local hospital and the Alma Mater Hospital of Antioquia.		planning stage; previous community trust; recognition of ancestral knowledge and the figure of the jaibaná; training in Spanish and Embera-Chamí.	delivery and full deployment.

Author	Year	Country	Ethnic group and language	Educational level	Sociodemographic situation	Objective	Methodological design and sample size (n)	Instruments	Type of health service	Barriers	Facilitators	Limitations
						care practices.						
**Gallardo-Peralta et al. [32]**	2021	Chile	Aymara and Mapuche individuals.<br><br>Language: Spanish	54% of the sample had incomplete basic education.	Older adults over 60 years old residing in rural areas of the regions of Arica and Parinacota and La Araucanía, Chile, participated.	To obtain reliability and factorial validity indicators of the Perceived Social Support Questionnaire (CASP) in a multiethnic sample of older Chilean adults, considering its use in Indigenous and non-Indigenous older adults.	Quantitative, instrumental/psychometric, cross-sectional, questionnaire validation study. Total sample (n=800) 231 non-Indigenous, 201 Aymara, and 368 Mapuche.	Sociodemographic questionnaire: age, sex, marital status, living arrangements, educational level, and affiliation. Perceived Social Support Questionnaire (CASP). Family Apgar.	It does not directly evaluate health services or healthcare access.	Rurality, belonging to Indigenous ethnic minorities, low schooling, illiteracy, potential vulnerability of older adults, and cultural differences in the way social support is understood.	Family networks as a central support in old age; support from spouse/partner, children, and other relatives; emotional, advisory, and instrumental support.	It does not directly evaluate health services or healthcare access. The study belongs to the field of psychological assessment, social gerontology, and family support.

**Note.* *Cells marked as "not reported," "not mentioned," or "not found" reflect information not available in the source extraction document.*
